# Impacts of ADHD Symptomatology on the Response to Cognitive-Behavioural Therapy with Gilles de la Tourette Syndrome Patients

**DOI:** 10.3390/jcm13102975

**Published:** 2024-05-18

**Authors:** Emmanuelle Mazur-Lainé, Houda Soubata, Julie B. Leclerc, Pierre J. Blanchet, Kieron P. O’Connor, Marc E. Lavoie

**Affiliations:** 1 Centre de Recherche de l’Institut Universitaire en Santé Mentale de Montréal, Montréal, QC H1N 3V2, Canada; emmanuelle.mazur-laine@umontreal.ca (E.M.-L.); houda.soubata@umontreal.ca (H.S.); leclerc.julie@uqam.ca (J.B.L.); pierre.j.blanchet@umontreal.ca (P.J.B.); kieron.oconnor@umontreal.ca (K.P.O.); 2 Département de Psychologie, Université de Montréal, Montréal, QC H2V 2S9, Canada; 3 Département de Psychologie, Université du Québec à Montréal, Montréal, QC H2X 3P2, Canada; 4 Groupe d’étude sur les Troubles Tic, d’Accumulation Compulsive et Obsessionnel-Compulsif (GE-tic-tac-toc), Montréal, QC H1N 3V2, Canada; 5 Centre de Recherche du CIUSSS du Nord-de-l’Île-de-Montréal, Montréal, QC H4J 1C5, Canada; 6 Faculté de Médecine Dentaire, Département de Stomatologie, Université de Montréal, Montreal, QC H3T 1J4, Canada; 7 Département de Psychiatrie et Addictologie, Université de Montréal, Montreal, QC H3T 1J4, Canada; 8 Département de Sciences Humaines, Lettres et Communication, Université TÉLUQ, Quebec, QC G1K 9H6, Canada

**Keywords:** tic disorders, chronic tics, CBT, YGTSS, CAARS

## Abstract

(1) **Background**: Gilles de la Tourette Syndrome (TS) is a neurodevelopmental disorder characterized by motor and vocal tics. Attention deficit and hyperactivity disorder (ADHD) is a common comorbidity of TS that adds further impairment. Cognitive-behavioural therapy (CBT) has shown efficacy in treating tics, yet its effectiveness in individuals with TS and comorbid ADHD remains unclear. Also, it is suggested that ADHD characteristics like executive dysfunction and inattention could hinder the response to CBT. This study aims to compare the response to CBT for tics and its maintenance six months post-therapy among TS individuals with and without ADHD symptoms. (2) **Methods**: In this study, 55 TS participants who completed 14-week CBT for tics were split into high (TS+) or low (TS−) ADHD symptomatology groups. Outcomes were evaluated using the Yale Global Tic Severity Scale (YGTSS) regarding global tic severity and motor and vocal tic frequency post-CBT and at a 6-month follow-up. (3) **Results**: No significant group difference was found regarding improvements post-CBT (*n* = 55), nor the maintenance six months later (*n* = 45). (4) **Conclusions**: ADHD symptoms may not hinder the response to CBT or its maintenance, suggesting that TS individuals with ADHD symptoms may not require specialized CBT interventions.

## 1. Introduction

### 1.1. Gilles de la Tourette Syndrome

Gilles de la Tourette Syndrome (TS) is a neurodevelopmental disorder within the family of tic disorders, characterized by the repetitive manifestation of non-rhythmic movements (motor tics) and vocalizations (vocal tics) [1,2]. Tics can be simple or complex. Simple tics are brief and meaningless sounds or movements, and complex tics involve meaningful speech or longer and coordinated movements [3]. According to the DSM-5 criteria [4], a TS diagnosis requires at least two different motor tics and one vocal tic. Additionally, tics must have been present for at least one year and begin before the age of 18 without being attributed to another medical condition or substance use [4]. However, cases of tics appearing after the beginning of adulthood have also been reported [5]. Tics are often preceded by a premonitory sensation described as physical discomfort or dissatisfaction, which disappears after the tic execution [6]. Chronic tic disorder is another tic disorder like TS, with the difference being that chronic tics are characterized by only one type of tic, either motor or vocal [4]. Since there is a significant overlap between the diagnostic criteria for TS and chronic tic disorder, and since they are often grouped, they will be referred to as “TS” in the current study. The prevalence of TS is estimated to be under 1% in children and decreases to approximately 0.05% in adults [7], as tics tend to decrease during adolescence [8,9,10]. Indeed, tics seem to reach their maximum severity around the ages of 10 to 12 [11]. Furthermore, this condition is approximately four-times more prevalent in men than women [2,12]. However, this gender difference may be explained by the fact that tics are usually less disruptive in women, making them less inclined than men to seek help and, thus, be diagnosed [13].

Physiological and neuroanatomical studies support the hypothesis of an anatomical and functional alteration in the cortico-striatal-thalamo-cortical (CSTC) network in individuals with TS [14]. These abnormalities lead to motor circuit hyperactivity and reduced inhibitory capacity [7]. Worbe et al. [15] identified a correlation between the severity of tics and abnormal brain connectivity profiles, particularly in fibres connecting primary motor and sensory areas with the basal ganglia and thalamus. This hypothesis aligns with findings suggesting that tics develop and persist due to altered dopaminergic activity [7]. Indeed, individuals with TS have a particular sensitivity to reinforcement learning, modulated by the brain’s dopaminergic system. The execution of tics is followed by a release of dopamine, which acts as a reinforcer and leads to the maintenance of tics [16]. The role of dopamine in TS is supported by studies demonstrating the effectiveness of tic management with treatment using typical antipsychotics that antagonize dopamine D2 receptors [7].

Pharmacotherapy is one of the treatment options for reducing tics. Antipsychotic medications that act as dopamine antagonists are used for this purpose, in line with findings suggesting that TS is associated with dopaminergic hyperactivity [17]. Moreover, according to Pringsheim et al. [18], antipsychotics may be even more effective for patients with tic disorders comorbid with ADHD, which brings us to our next question, about the impact of ADHD on the outcome of TS symptoms following treatment.

### 1.2. Impact of Comorbidities in Gilles de la Tourette Syndrome

Comorbidities are present in approximately 90% of cases of tic disorders [19,20], and they generally worsen the quality of life of those affected [21]. It is not uncommon for more than one comorbidity to be present, and behavioural problems increase with the number of comorbidities [22]. ADHD is one of the most common comorbidities of tic disorders, occurring in approximately 60% of TS cases [7,22] but only in 2 to 12% of the general population [2,10,20,23]. ADHD is a neurodevelopmental disorder characterized by inattentive symptoms, hyperactivity, and impulsivity [4]. This condition is frequently accompanied by deficits in executive functions, including working memory, planning, vigilance, monitoring, and response inhibition [24,25]. The severity of ADHD symptoms is the strongest predictor of quality of life in TS accompanied by ADHD in children and can lead to more anxiety, mood disorders, aggression, behavioural problems, adaptation difficulties, and psychosocial stress [7,26]. These impacts ultimately result in a more significant impairment in functioning than tics alone [7,27]. For these reasons, it is crucial to differentiate between tic disorders alone and tic disorders with substantial ADHD symptomatology, both in terms of diagnosis and treatment outcomes.

### 1.3. Cognitive and Behavioural Therapies for Gilles de la Tourette Syndrome

Cognitive-behavioural therapy (CBT) has proven effective in improving TS symptoms. CBT is a psychotherapy that uses cognitive and behavioural strategies to change actions or modify factors that appear to lead to the targeted behaviour to ultimately alter, replace, or eliminate the behaviour [28]. In treating tic disorders, CBT focuses on identifying premonitory urges and replacing tics with alternative responses. A systematic review by Pringsheim et al. [18] supports using CBT to treat tics. The authors also emphasize the importance of considering comorbidities when treating tic disorders. Habit Reversal Therapy (HRT) is a similar form of CBT used for treating tics, based on the idea that symptoms are developed through conditioning. HRT offers several behavioural strategies to break the conditioning chain, especially the replacement of tics by an incompatible motor action [29]. Another critical component of any therapy is tic awareness. It involves helping the patient to identify their tics and the contexts in which they occur and to detect the premonitory urges that precede them [30]. Since the introduction of psychotherapies for tics in the 1970s, we know that this step is crucial since some symptoms develop without the patient’s awareness [31].

In the current research, we propose using the cognitive-behavioural and psychophysiological (CoPs) intervention, which represents another specialized version of CBT. In that therapy, a different intervention framework considers tics a behavioural response to a gradual increase in muscle tension and sensorimotor activation [32,33,34]. Furthermore, one of the suggested mechanisms of this treatment is improving motor control concerning the high sensorimotor activation observed in these patients. Specifically, the CoPs approach makes it possible to target sensorimotor processes and not only the antagonistic actions of the muscles in connection with the generation of tics. This intervention improved motor performances at the Purdue Pegboard [35] and induced changes in movement-related cortical activity [36,37,38], suggesting that these intervention strategies make it possible to improve the symptoms of TS and overactivity. Recent data suggest that the CoPs therapy is on par with other gold-standard treatments of TS, such as CBIT [39]. Cognitive Behavioural Intervention for Tics (CBIT) is a similar approach that combines HRT with other behavioural strategies. Among these, we find psychoeducation, which consists of learning about tic disorders. Generalization training involves putting into practice the techniques learned in various contexts. The function-based assessment is used to identify the chain of antecedents and consequences surrounding ICTs. Finally, the function-based intervention involves establishing strategies to minimize the adverse effects of tics [30].

A meta-analysis by Shou et al. [40] described the response to CBT in people with tic disorders based on 12 studies involving 536 participants. The experimental groups underwent various forms of CBT, such as HRT, CBIT, and anger control training. The control groups received other therapies, such as relaxation and psychoeducation, primarily aimed at managing stress and anxiety. The results indicate that CBT was more effective than other interventions in reducing the severity of motor tics, but no significant difference was found in vocal tics. A study by Greenberg et al. [41], comparing the effectiveness of a new adaptation of CBIT to traditional CBIT in children and adolescents, indicated that the severity of tics, tic-related impairment, and ADHD symptoms decreased for both therapy groups. Three months later, the improvement remained stable across the first post-treatment and evaluation assessments. Similarly, a meta-analysis by Frey and Malaty [42] shows that the improvement following behavioural therapies for tics tends to be maintained for at least 2 months up to 2 years.

It has been found that CBT aiming to improve organization, planning, distractibility, and procrastination can improve inattention, impulsiveness, hyperactivity, working memory, inhibition, self-monitoring, depression, and anxiety among unmedicated adults with ADHD [43]. However, it is unclear if this population responds well to therapies aiming specifically on tic reductions without targeting ADHD components. Indeed, some data suggest that ADHD characteristics may hinder the response to CBT. Executive functions, like flexibility, planning, working memory and response inhibition, could play a role in understanding and applying the principles of CBT [44,45]. In CBT for tics specifically, response inhibition and monitoring, which are more impaired in individuals with TS and ADHD than in those with TS alone, are essential in the ability to suppress tics voluntarily [44]. Indeed, Chang et al. [25] found that children with better working memory and response inhibition abilities tend to be more responsive to behavioural therapy. Earlier findings also underlined that reduced attentional skills, one of the main symptoms of ADHD, are associated with greater difficulty in inhibiting tics [46]. Therefore, people with executive dysfunction and attention deficits might need a more individualized approach that considers those difficulties [45]. More specifically, CBT may not be as beneficial for people with TS and comorbid ADHD symptoms as for those with TS only.

CBT aiming at tic reduction is well documented in the literature and generally yields positive responses. However, data regarding the response to CBT for tics in individuals with primary TS in conjunction with ADHD symptoms, compared to those with only TS, are scarce and equivocal. Furthermore, to our knowledge, the maintenance of the effects of treatment in participants with TS and ADHD has not been assessed beyond three months, nor has it been compared to a paired TS-only group.

### 1.4. Objectives and Hypotheses

Given the variability in earlier research regarding the influence of ADHD symptoms on the response to CBT for tics and the additional challenges faced by individuals with comorbid TS and ADHD, further investigations are needed among TS and ADHD populations regarding their clinical responses to CBT. The main goal of the current study is to determine whether CBT leads to a different outcome in individuals with TS and ADHD symptomatology compared to those with TS alone in terms of tic severity or frequency. The first hypothesis is that, for those symptoms, CBT will be less beneficial for individuals with TS + ADHD symptoms than for those with TS only. We also investigate whether these clinical groups’ post-therapy effects remain stable. Hence, the second hypothesis is that the maintenance of the response six months after the therapy will be stronger among individuals with TS only compared to TS with comorbidities.

## 2. Materials and Methods

### 2.1. Participants

The data for the current study come from the Laboratoire de psychophysiologie cognitive et sociale at the Centre de recherche de l’Institut Universitaire en Santé mentale de Montréal (CR-IUSMM). The current project is also part of a larger project approved by the local ethics review board to assess the effect of comorbidity and CBT in Tourette syndrome. Participants were selected from the database of an open trial evaluating the effectiveness of a CoPs intervention for tics among people with TS or chronic tic disorder [47]. For the original study, participants were screened by telephone, followed a semi-structured interview, a semi-structured evaluation and a neurological screening, and were evaluated on tic severity and characteristics by an independent psychologist. Participants were excluded if they had a major medical history, a head injury with sensorimotor impairment, autism, an IQ below 75, an axis I or II psychiatric disorder requiring treatment or a neurological problem, and if they were already following a therapy, were misusing alcohol or drugs, or were treated with a psychotropic drug not relevant to TS or ADHD. Out of the 236 people who were assessed for eligibility, 85 participants met the inclusion criteria and followed the intervention, all of whom had been diagnosed with TS or a tic disorder and had been assessed on anxiety and depression prior to the intervention.

**Figure 1:**
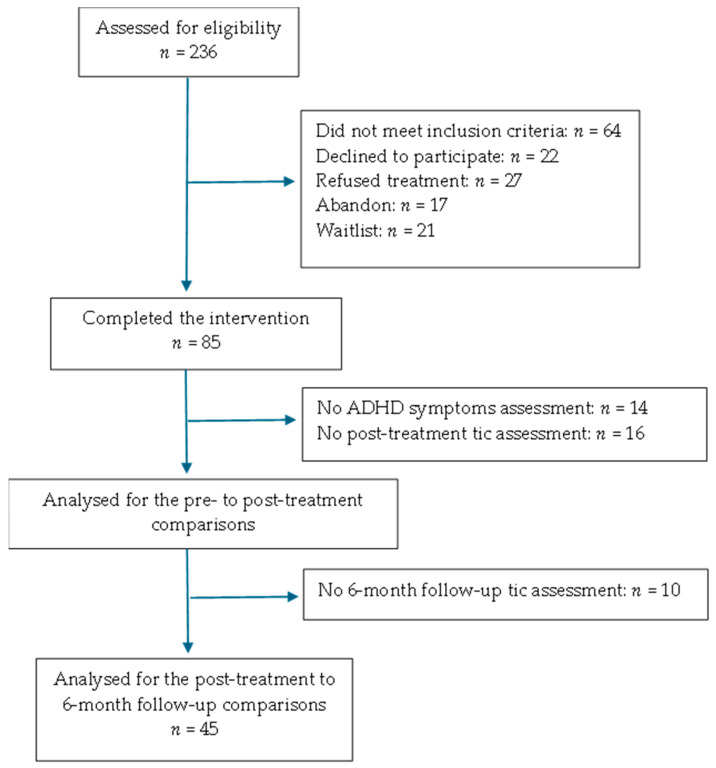
Participant flow chart.

For the current study, TS (or chronic tics) participants were selected among the initial 85 participants if they had been evaluated for symptoms of ADHD and if they had completed the pre- and post-therapy tic evaluations. Thus, 55 participants met those criteria, 45 of which had also completed the 6-month follow-up assessments, and 28 had completed the 1-year follow-up. The 16 TS participants who had not completed the post-treatment tic assessments were dropped from all analyses, and the one-year follow-up scores were not used due to insufficient sample size. We also selected 43 control participants with no history of tic disorder, with or without ADHD symptomatology. The final sample contained 98 participants (43 controls and 55 TS). The patient and control groups were split in half based on the CAARS global scores to constitute four groups based on TS and ADHD symptomatology: TS with ADHD symptomatology (TS+), TS without ADHD symptomatology (TS−), control with ADHD symptomatology (control+), and control without TS and ADHD symptomatology (control−). Regarding ADHD medication, 1 control− participant used Adderall, 3 TS− participants used Adderall or Strattera, and 2 TS+ participants used Adderall or biphentin. It should be noted that the control participants were used only to verify the correspondence between the groups on baseline demographic and clinical variables and were not included in the repeated measures analyses since they did not experience any treatment. A flow chart is shown in Figure 1.

### 2.2. Material

***Yale Global Tic Severity Scale (YGTSS, 1989).*** The YGTSS is a tool that aims to assess the severity of tics and is administered through a semi-structured interview [48]. It is divided into five subscales rated from 0 to 5 (number, frequency, intensity, complexity, and interference of tics) and an impairment subscale rated from 0 to 50. The version used for this study was the 1992 edition, in which all six subscales contain scores for both motor and phonic (vocal) tics. The first five subscales are also divided into two categories: the tics as they present at their worst and the state of the tics at the time of evaluation. The latter was retained for this study. The YGTSS yields scores for the severity of motor tics, phonic tics, and overall tics, as well as a deterioration score and an overall score. Higher scores indicate greater severity. For this study, the selected measures are the global tic severity score, the motor tics frequency score, and the phonic tics frequency score. The test–retest reliability of the YGTSS was assessed at 0.84 [49]. Storch et al. [50] reported excellent internal consistency (α = 0.93–0.94), excellent temporal stability (ICC = 0.89) for the global severity score, and good discriminant validity with ADHD. However, these results were calculated from samples of children and adolescents, and the psychometric properties have not yet been tested with an adult sample.

***Conners’ Adult ADHD Rating Scales—Self Report, Short Version (CAARS-S:S; 1999).*** The CAARS is a questionnaire developed to assess ADHD symptoms in adults. The CAARS-S:S is a shorter self-report version, comprising 26 items rated on a Likert scale ranging from 0 (not at all) to 3 (very much/very frequently) [51]. It contains four subscales developed from factor analyses (inattention/memory, hyperactivity, impulsivity, and self-concept), a total ADHD index score, which represents the likeliness of the responder having ADHD, and a total score based on the two DSM criteria for ADHD (i.e., inattention and hyperactivity-impulsivity). A higher score is associated with more severe symptoms. Erhardt et al. [52] reported good test–retest reliability for the four subscales (0.80–0.91) and very good internal consistency (α = 0.86–0.92). However, a literature review suggests limited convergent validity (0.42–0.75) [53]. Given that CAARS is unsuitable for diagnosing ADHD, participants in this study are classified based on the presence of ADHD symptomatology rather than an official ADHD diagnosis. Individuals aged 17 and below were assessed using the Conners’ ADHD Rating Scale (Conners) [54], the children’s version of the CAARS. The measure used for the principal analyses is the total score since it reflects ADHD as described in the DSM-5.

***Beck Anxiety Inventory (BAI, 1988).*** The BAI (Beck Anxiety Inventory), developed by Beck et al. in 1988 [55], is a self-report questionnaire aiming to assess anxiety. It consists of 21 items, each addressing a specific anxiety symptom that the person being assessed must rate on a scale from 0 (not at all) to 3 (severely), indicating the degree to which they have been bothered by that symptom in the past month. The BAI demonstrates excellent internal consistency (α = 0.92) and moderate test–retest reliability, with a coefficient of 0.75.

***Beck Depression Inventory (BDI, 1961).*** The Beck Depression Inventory (BDI) is a self-report questionnaire used to measure depressive symptomatology. It consists of 21 items rated on a scale from 0 (not at all) to 3 (severely). The BDI demonstrates good internal consistency (α = 0.87), and its test–retest reliability is reported to range from 0.60 to 0.90 (M = 0.75) over intervals of 7 days to 4 months in non-clinical samples [56].

### 2.3. Procedure

In the current sample, all participants had previously been assessed for ADHD symptomatology (CAARS), anxiety (BAI), and depression (BDI) levels. Participants diagnosed with TS or chronic tics were also evaluated for the severity and frequency of their tics (YGTSS) before undergoing a 14-week CBT-CoPs intervention for tics. Tic severity and frequency were reassessed at the end of the treatment, and six months and one year later. The participants were accompanied by a certified psychologist with 5 to 7 years of experience specializing in CBT for tic disorders. They were first evaluated on tic severity, tic characteristics, the impact of tics on their life, their style of planning and thinking, and their beliefs about tics. They then discussed the goals with the therapist, were offered advice on how to deal with stigma, and received psychoeducation. The participants learned how to observe and describe their tics and premonitory urges, and how to identify contexts associated with tic onset. Those observations were reported in a daily diary. Finally, they learned physical and psychological techniques to prevent or reduce the physical tension that precedes tic bursts, to improve their muscle control, to develop better styles of action, and to regulate their emotions efficiently. All participants received 10 sessions at a rate of 1 session per week. The therapists followed the manual “Managing tic and habit disorders: A cognitive psychophysiological treatment approach with acceptance strategies” written by O’connor, Lavoie, and Schoendorff [32].

### 2.4. Data Preparation

Some participants had completed the BAI, BDI, and YGTSS twice before the treatment, during a waitlist period. The Reliable Change Index (RCI) method [57] was used to establish a single pre-treatment score for each measure. The RCI relies on the test–retest reliability coefficient of measurement to establish a threshold indicating which minimal variance in a participant’s scores can be considered a clinical change. In this context, it was used to determine whether each participant’s two pre-treatment scores were close enough to be regarded as clinically equivalent. For the YGTSS, RCI was applied for each of the 12 items, using a test–retest reliability coefficient of 0.84 [49] and a 90% confidence interval. For each item, participants whose variance between the two pre-treatment scores was sufficiently low were assigned the average of their two scores, while participants with high variance were assigned their second pre-treatment score, which was temporally closer to the onset of treatment. The total YGTSS scores were recalculated with the corrected item values. A total of 14 participants had changes in their YGTSS scores. The same procedure was conducted for the total scores of the BAI and BDI, with a test–retest reliability coefficient of 0.75 for both. Changes were made for seven participants for the BAI scores and one participant for the BDI scores.

The participants were divided based on their ADHD symptomatology, as determined by the median of the CAARS total scores. The median score was calculated for both the controls and the TS participants so that TS symptoms, which might impact CAARS scores, would not influence the division of the control participants. TS participants who scored equal or higher to the median of their group (M = 32) were classified into the TS+ group (*n* = 28), and the remaining TS participants were classified into the TS− group (*n* = 27). Control participants (M = 19) were separated in the same way to form a control− group (*n* = 19) and a control+ group (*n* = 24).

### 2.5. Statistical Analyses

**Preliminary analyses.** Preliminary analyses assessed the groups’ equivalence on demographic and clinical variables at baseline. The sex ratios and the age distributions between the four groups were compared with a chi-square test and an ANOVA, respectively. To take into consideration the possible impact of anxiety and depression on the response to treatment, since both disorders can influence tic severity and duration [58], BAI and BDI scores were compared across the groups using ANOVAs. The CAARS total scores were compared with an ANOVA to confirm that the median splits allowed for sufficient ADHD symptomatology differences between the groups. All post hoc comparisons were made with Tukey’s correction for multiple comparisons, except for assumption violation.

**Main analyses.** Since there was attrition at every measurement point, separate analyses were conducted to answer the two aims and preserve all available data for the first aim. First, the responses to CBT in the TS− (*n* = 27) and TS+ (*n* = 28) groups were compared using an analysis of covariance (ANCOVA), with the groups as the independent variable, the post-treatment tic scores as the dependent variable, and the baseline tic scores as a covariate to consider any baseline variability across groups. This analysis was repeated separately for the three YGTSS scores (global tic severity score, motor tic frequency score, and phonic tic frequency score). Secondly, to assess whether the maintenance of the response over time was impaired by ADHD symptomatology, ANCOVAs were performed with post-treatment scores as a covariate, 6-month follow-up scores as a dependent variable, and group as an independent variable for the three tic scores separately. The second analyses were conducted only with the 45 TS participants who had completed the post-treatment and the 6-month follow-up YGTSS assessments (TS−: *n* = 21; TS+: *n* = 24). All analyses were conducted in RStudio, version 4.2.3.

**Assumptions.** Corrections for outliers were considered for BAI and BDI scores but not for YGTSS and CAARS scores because the current study focuses on TS and ADHD clinical samples. The threshold for outliers was set at z = 3.29 (CI = 95%). Variables were considered abnormal if the skewness or kurtosis indices exceeded ±2, but skewness beyond this threshold was not regarded as problematic if all groups had a distribution leaning in the same direction [59]. For the ANOVAs, homoscedasticity was evaluated using Tabachnick and Fidell’s method [60]. For the variables that did not meet the criterion for normality and homogeneity, the analyses and post hoc comparisons were made with robust ANOVA models [61,62]. All inter-subject scores were treated as independent, following the independence of scores assumption. For the ANCOVAs, the criterion for linearity was linear-looking regression plots for each group individually [60]. The normality of the regression residuals was evaluated with the same criteria as the ANOVA. The homoscedasticity of the regression line for each group was assessed by verifying if the residuals’ plot formed a rather oval-like distribution. The regression slopes were considered homogeneous if no interaction was found between baseline scores and the group variable, as assessed by an ANOVA test. A non-parametric robust ANCOVA model was used for the variables that did not meet these assumptions, which evaluates if the groups are statistically different at different covariate values [61].

## 3. Results

### 3.1. Preliminary Analyses

Table 1 presents the results of group comparisons on demographic and clinical variables. Biological sex ratios (F/M) were similar across all groups (χ^2^[3] = 3.94, *p* = 0.27). Ages were also similar across groups (F[3, 94] = 1.26, *p* = 0.29) and ranged from 12 to 60 across the entire distribution (control−: 19–60; control+: 20–55; TS−: 12–56; TS+: 14–60). One TS+ participant scored a high BDI (47, z = 4.63). The score was removed from the BDI variable, but the participant was retained to maintain the sample size. There were no outliers on the BAI, CAARS, or the three YGTSS scores. The BAI and BDI distributions did not meet the normality assumption criterion for kurtosis (BAI: control− = 5.66; BDI: control− = 6.49, control+ = 4.78), so a robust ANOVA model was used to analyse these variables.

The scores of the BAI differed significantly between groups (F[3, 94] = 7.34, *p* = 0.002), and the control− group showed lower scores than the three other groups. The groups also differed on the BDI scores (F[3, 93] = 15.51, *p* < 0.001): the control− participants scored lower than the three other groups, and the control+ scored lower than the TS+. The comparisons on the CAARS confirmed a between-groups difference (F[3, 94] = 81.78, *p* < 0.001). The four groups were all statistically different, the TS+ group having the highest mean score, followed by the control+, the TS−, and the control−.

### 3.2. Main Analyses

Table 2 shows the results of the analysis of covariance for the group effect on post-treatment global tic severity (YGTSS total), frequency of motor tics (YGTSS-2a), and frequency of phonic tics (YGTSS-2b), controlling for baseline tic severity scores. For the post-therapy total tic severity scores, an effect of baseline tic severity was found (F[1, 52] = 35.80, *p* < 0.001), but no group effect was revealed (F[1, 52] = 2.44, *p* = 0.12). The same tendency was found for vocal tic frequency (baseline score: F[1, 52] = 75.44, *p* < 0.001; group: F[1, 52] = 0.12, *p* = 0.74). The motor tic frequency score distribution did not meet the assumptions of linearity and homoscedasticity, so a non-parametric ANCOVA model was used. The model compared the groups on three covariate values (3, 4, and 5), none of which yielded significant differences.

Table 3 presents the results of the post-treatment and 6-month follow-up group comparisons. A non-parametric model was used to assess the change in total tic severity due to the violation of linearity, normality, and homoscedasticity. Five post-treatment tic severity values were selected (11, 13, 18, 21, and 25), and none had a significant difference between the groups regarding the 6-month follow-up scores. A robust model was also used for the frequency of phonic tics, with two points of the covariate for comparison (0 and 1). Neither had a significant difference on the 6-month scores; the same tendency was found. Finally, there was no between-groups difference in the frequency of motor tics six months after therapy (F[1, 42] = 0.15, *p* = 0.705), but there was an impact of post-treatment motor tic frequency (F[1, 42] = 39.81, *p* < 0.001).

## 4. Discussion

Our goal was to clarify whether CBT impacts individuals with TS + ADHD differently compared to those with TS alone in terms of tic severity or frequency. The first hypothesis was that CBT would be less beneficial for individuals with TS + ADHD symptoms than for those with TS only. As a second step, we also proposed that the maintenance of the response six months after the therapy would be stronger among individuals with TS only compared to TS + ADHD. The first hypothesis was not confirmed. Indeed, no difference was found between the two groups regarding reductions in tics following the intervention regarding the severity and frequency of motor or phonic tics. The second hypothesis was also rejected since the trajectory of tic severity and frequency at the 6-month follow-up did not differ between the two groups.

These results could mean that ADHD symptoms in the TS+ group did not necessarily interfere with their ability to inhibit their tics and maintain those abilities after therapy, contrary to the idea that attention difficulties and executive dysfunction could hinder the ability to suppress tics. However, the separation criterion for the two TS groups was the CAARS total score, which considers all the symptoms assessed by the questionnaire: inattention, memory problems, hyperactivity, restlessness, impulsivity, and emotional lability. Since these symptoms may impact the response to CBT in different ways and to a different extent, the results might have been more revealing if we had conducted separate analyses to isolate their respective effect on CBT.

CBT appears equally effective in individuals with comorbid ADHD symptoms, which matches the findings of Greenberg et al. with an adolescent population [41]. The latter study proposed a modified therapy based on the original CBIT protocol proposed by Piacentini et al. [29] and an adapted CBT for ADHD in adolescents proposed by Sprich et al. [63]. Our current CBT, the so-called Cognitive Psychophysiological therapy (CoPs) proposed by O’Connor et al., was validated with an adult population [64]. Within that approach, cognition interacts with physiological factors such as an increased sensorimotor activation, leading to over-reactivity (e.g., overusing specific muscles and exerting more effort than necessary for a task), acting as a circular linking to tic onset [47]. The CoPs model considers the release of tension as a part of a regulation system characterized by sensorimotor functioning that tends to increase muscular activation and tension. These features are potentially shared with the ADHD and TS population, which explains why our TS+ group benefitted the therapy as well as the TS− group. Thus, ADHD symptoms do not appear to impair post-therapy recovery in the TS+ population.

Lastly, the temporal stability of the scores across the 6-month follow-up indicates that the effects persist after the completion of therapy, aligning with what was found for a three-month follow-up [41]. The results regarding the impact of pre-treatment tic scores on post-treatment tic scores and the effect of post-treatment on 6-month follow-up tic scores imply a relationship between the symptom evolution at different time points across participants.

The TS+ group had higher baseline anxiety and depression scores, although no significant difference was found with the TS− group. Whether the higher global tic severity baseline scores in the TS+ group are better explained by the higher ADHD symptomatology or by the higher anxiety scores is uncertain. Indeed, anxiety is known to enhance tics [65,66,67]. The equal level of improvement between the TS groups could, therefore, be explained by a similar decrease in anxiety, rather than by a lack of interference with ADHD symptoms. Although the evolution of anxiety was not evaluated in the current study, the initial trial from which the data were taken reported an improvement in anxiety and depression after CBT, which could also apply to the current sample. Furthermore, CBT for ADHD has been shown to reduce depression and anxiety.

Our results align with previous findings showing that different types of CBTs can significantly reduce the total tic disorder score and motor tic score in individuals with TS in various age groups [40]. However, our results revealed an improvement in vocal tics, which was not corroborated in the meta-analysis of Shou et al. [40].

## 5. Limitations and Future Orientations

Further studies should compare the responses to CBT of higher and lower executive functioning participants. Furthermore, the CAARS total score is not a clinical diagnosis of ADHD and, therefore, our sample more closely represents a group with ADHD symptomatology rather than a group diagnosed with ADHD. The results are, however, still relevant since a considerable proportion of the TS population presents subclinical ADHD symptoms.

It is also assumed that our results could be impacted by the sample’s unusually high female-to-male ratio, approximately 50–50. Indeed, the TS and ADHD populations are predominantly male. Although more research is necessary to establish the gender differences in the treatment of tic disorders with comorbid ADHD specifically, it appears that both disorders present in different ways depending on gender [68,69]. Given these differences, it is possible that men and women with TS− and TS+ react differently to CBT for tics. Gender differences were not considered in the current study but should be investigated in future research. From another perspective, although the proportions of our sample do not reflect those in the TS− and TS+ populations, they allow for greater representation of women in tic disorders and ADHD research. Indeed, current and past studies are often conducted on male-dominant samples, generating results that are not necessarily applicable to the women’s side of the spectrum.

Finally, the sample sizes for post-treatment and six-month follow-up measurements are limited. Many participants had to be excluded from the start because they had other disorders, such as OCD, habit disorders, pronounced anxiety, and depressive symptoms. This loss of participants reduced statistical power, increasing the chances that the analyses may not have detected some fundamental differences between the study populations.

## 6. Conclusions

The results suggest that CBT adapted for tics may yield the same response among people with TS, whether they present significant ADHD symptomatology or not. Indeed, no difference was found in the improvement following CBT and its maintenance over six months between people who have TS with ADHD symptomatology and people with TS only. The similar response across groups could mean that ADHD symptoms do not hinder the response to CBT for tics and its maintenance over time. Therefore, individuals with TS and ADHD symptomatology may not necessarily require an adapted CBT approach for their tics or ADHD. These findings allow for a broader understanding of how comorbid ADHD could interfere with TS treatments and can guide clinicians in the process of choosing adequate interventions for TS patients while taking comorbidities into account. However, this issue needs further investigation with a more extensive clinical ADHD sample and a consideration for gender differences, the role of anxiety in tic reduction following CBT, and the respective impacts of the different ADHD symptoms and executive function impairments that characterize this disorder.

## Figures and Tables

**Table 1:** Preliminary comparisons on demographic and clinical variables.

	Ctrl− (*n* = 19)	Ctrl+ (*n* = 24)	TS− (*n* = 27)	TS+ (*n* = 28)	Results
Age	39.00 (12.82)	32.92 (9.58)	33.26 (12.20)	33.39 (12.03)	F[3, 94] = 1.26, *p* = 0.292
Sex (F:M)	7:12	11:13	15:12	18:10	χ^2^[3] = 3.95, *p* = 0.268
Anxiety (BAI)	2.66 (3.47)	6.71 (4.73)	7.26 (7.31)	9.64 (7.04)	F[3, 94] = 7.34, *p* = 0.002 Ctrl− vs. Ctrl+: t = −4.62, *p* = 0.002 Ctrl− vs. TS−: t = −3.12, *p* = 0.004 Ctrl− vs. TS+: t = −6.73, *p* < 0.001
Depression (BDI)	2.55 (4.09)	5.60 (6.04)	8.37 (6.77)	12.48 (8.20)	F[3, 93] = 15.51, *p* < 0.001 Ctrl− vs. Ctrl+: t = −3.17, *p* = 0.02 Ctrl− vs. TS−: t = −5.75, *p* < 0.001 Ctrl− vs. TS+: t = −9.93, *p* < 0.001 Ctrl+ vs. TS+: t = −6.76, *p* < 0.001
ADHD symptomatology (CAARS)	10.37 (5.71)	28.50 (8.12)	20.11 (6.18)	42.14 (8.35)	F[3, 94] = 81.78, *p* < 0.001 Ctrl− vs. Ctrl+: t[94] = −8.11, *p* < 0.001 Ctrl− vs. TS−: t[94] = −4.47, *p* = 0.001 Ctrl− vs. TS+: t[94] = −14.69, *p* < 0.001 Ctrl+ vs. TS−: t[94] = 4.11, *p* < 0.001 Ctrl+ vs. TS+: t[94] = −6.74, *p* < 0.001 TS− vs. TS+: t[94] = −11.22, *p* < 0.001

BAI: Beck Anxiety Inventory, BDI: Beck Depression, ADHD: attention deficit and hyperactivity disorder, CAARS: Conners’ Adult ADHD Rating Scale, Ctrl−: control participants without Gilles de la Tourette Syndrome and ADHD symptomatology, Ctrl+: control participants with ADHD symptomatology, TS−: Gilles de la Tourette Syndrome without ADHD symptomatology, TS+: Gilles de la Tourette Syndrome with ADHD symptomatology, BAI TS+: *n* = 27. The significance threshold is *p* ≤ 0.05. For the post hoc comparisons, only the significant results are shown.

**Table 2:** Results of the analyses on the pre-treatment and post-treatment YGTSS scores.

	Time	TS− (*n* = 27)	TS+ (*n* = 28)	Results
Global tic severity (YGTSS total)	Pre	30.96 (13.67)	37.48 (17.11)	Pre: F[1, 52] = 35.80, *p* < 0.001 Group: F[1, 52] = 2.44, *p* = 0.124
	Post	22.20 (11.96)	21.45 (10.70)	
Frequency of motor tics (YGTSS-2a)	Pre	3.59 (1.19)	3.61 (1.47)	3: F = 1.97, *p* = 0.069 4: F = 1.92, *p* = 0.066 5: F = 1.97, *p* = 0.066
	Post	2.44 (1.37)	2.79 (1.47)	
Frequency of phonic tics (YGTSS-2b)	Pre	1.37 (1.75)	1.91 (1.86)	Pre: F[1, 52] = 75.44, *p* < 0.001 Group: F[1, 52] = 0.12, *p* = 0.735
	Post	0.81 (1.27)	1.04 (1.32)	

YGTSS: Yale Global Tic Severity Scale, TS−: Gilles de la Tourette Syndrome without ADHD symptomatology, TS+: Gilles de la Tourette Syndrome with ADHD symptomatology. The significance threshold is *p* ≤ 0.05.

**Table 3:** Results of the analyses on the post-treatment and 6-month follow-up YGTSS scores.

	Time	TS− (*n* = 21)	TS+ (*n* = 24)	Results
Global tic severity (YGTSS total)	Post	22.20 (11.96)	21.45 (10.70)	11: F = 0.22, *p* = 0.833 13: F = 0.03, *p* = 0.976 18: F = 0.26, *p* = 0.798 21: F = 1.16, *p* = 0.266 25: F = 0.92, *p* = 0.377
	6 months	22.36 (10.74)	21.31 (12.33)	
Frequency of motor tics (YGTSS-2a)	Post	2.44 (1.37)	2.79 (1.47)	Post: F[1, 42] = 39.81, *p* < 0.001 Group: F[1, 42] = 0.15, *p* = 0.705
	6 months	2.76 (1.48)	2.79 (1.61)	
Frequency of phonic tics (YGTSS-2b)	Post	0.81 (1.27)	1.04 (1.32)	0: F = 1.12, *p* = 0.291 1: F = 0.71, *p* = 0.487
	6 months	1.10 (1.26)	0.96 (1.52)	

YGTSS: Yale Global Tic Severity Scale, TS−: Gilles de la Tourette Syndrome without ADHD symptomatology, TS+: Gilles de la Tourette Syndrome with ADHD symptomatology. The significance threshold is *p* ≤ 0.05.

## Data Availability

The algorithms that were used for this study are available at https://github.com/emmanuelleml/PSY3008-GTS-ADHD, accessed on 21 February 2024.
